# Prevalence and influencing factors of chronic pain in middle-aged and older adults in China: results of a nationally representative survey

**DOI:** 10.3389/fpubh.2023.1110216

**Published:** 2023-04-17

**Authors:** Zhonghua Ai, Churou Tang, Puxian Peng, Xuan Wen, Songyuan Tang

**Affiliations:** ^1^Institute of Health Studies, School of Public Health, Kunming Medical University, Kunming, Yunnan, China; ^2^Department of Biology, University of Rochester, Rochester, NY, United States; ^3^School of Public Health, Kunming Medical University, Kunming, Yunnan, China

**Keywords:** chronic pain, prevalence, influencing factors, middle-aged and older adults, China

## Abstract

**Background:**

With China's rapidly aging population, chronic pain has become a major public health issue. This article aims at determining associations between chronic pain and multiple factors, including demographic characteristics, health status, and health service utilization of middle-aged and older adults in China.

**Methods:**

We selected all the 19,829 respondents who were over 45 years old from the China Health and Aging Tracking Survey 2018 (CHARLS) as our study population. The key information in terms of the body pain, demographic characteristics, health status, behaviors and health services use was extracted and analyzed. Logistic regression model was used to determine the influencing factors of chronic pain.

**Results:**

Analysis revealed that 60.02% (9,257) of the data from this survey reported physical pain, with pain sites concentrated at the head (40.9%), lower back (62.2%) and knees (47.2%). Pain was positively associated with influencing factors for pain: being a female (OR = 2.10, 95% CI 1.90–2.33, *p* < 0.001), living in a western region (OR = 1.28, 95% CI 1.16–1.41, *p* < 0.001), living in a rural area (OR = 1.14, 95% CI 1.06–1.23, *p* < 0.001), smoked (OR = 1.26, 95% CI 1.14–1.38, *p* < 0.001), drank alcohol (OR = 1.16, 95% CI 1.06–1.26, *p* = 0.001), and had poor self-rated health (OR = 6.84, 95% CI 5.41–8.65, *p* < 0.001), had hearing problems (OR = 1.23, 95% CI 1.11–3.37, *p* < 0.001), were depressed (OR = 1.56, 95% CI 1.03–1.29, *p* < 0.001), had arthritis (OR = 2.21, 95% CI 2.02–2.41, *p* < 0.001), stomach disorders (OR = 1.69, 95% CI 1.55–1.85, *p* < 0.001), visited a Western medicine hospital (OR = 1.28, 95% CI 1.10–1.50, *p* = 0.002), and visits to other medical institutions (OR = 1.42, 95%CI 1.22–1.64, *p* < 0.001). On the other side, as a protective factor for pain, having nighttime sleep ≥7 h (OR = 0.74, 95%CI 0.68–0.80, *p* < 0.001) was negatively associated with pain.

**Conclusion:**

Physical pain affects many older adults. Women, regional, rural residents, smokers, alcohol drinkers, people with poor self-rated health, those having <7 h of sleep at night, those with hearing problems, depression, arthritis, stomach disorders, and people who visits Western hospitals or other medical institutions are at greater risk for pain and deserve the attention of health care providers and policy makers to focus on pain prevention and management in middle-aged and older adults. Future research studies should also focus on the impact of health literacy on pain prevention and management outcomes.

## Introduction

The prevalence of chronic pain in China is increasing yearly, affecting an increasing number of middle-aged and older adults people (1). Chronic pain affects people worldwide and has become an inevitable problem (2). Chronic pain is usually defined as the continued presence of chronic pain for more than 1 month after some tissue damage has subsided, or recurrent chronic pain for more than 3 months. Chronic pain can have a serious disturbance in sleep and metabolism. Furthermore, a long course of chronic pain may lead to depression, disability, and a decreased in quality of life (3). By 2025, China is expected to have 20% of its population aged 60 years and older (4). An aging population will undoubtedly increase the burden on society, especially on the healthcare system (5). Due to the current predicament in China, sufficient attention to chronic pain management for middle-aged and older adults people is required. The Health China Action (2019–2030) aims to maintain the health of middle-aged and older adults people in all aspects and strive to improve their quality of life (6).

In the certain socio-cultural environment, chronic pain is considered less important in healthcare. Most people believe that the rate of chronic pain increases with age (7). In addition to multiple fatal complications, chronic pain can cause great suffering to patients (8, 9). Chronic pain also imposes a huge global economic burden. For example, European countries alone are conservatively estimated to have invested $185 billion in chronic pain management in 2014 (10). Moreover, chronic pain affects both the physical and mental health of middle-aged and older adults people, and has a significant impact on society (11).

Therefore, it is necessary to understand the prevalence of chronic pain in middle-aged and older adults people in China and the factors affecting it, thus, to provide a basis for health policymakers and implementers. Although a growing concern about the dangers of chronic pain in older adults exists, relationships between chronic pain and other health-related factors are unclear. In this study, we focus on determining relationships between chronic pain and demographic characteristics, health status, and health behaviors in adults aged 45 years and older, providing information that could contribute to the Health China Initiative (2019–2030) program.

## Materials and methods

### Data sources and sample

In our cross-sectional study, the data was extracted from the China Health and Retirement Longitudinal Study (CHARLS) database, which aiming to study the issues arising from aging in China and promote corresponding researches (12). The CHARLS study used a multistage stratified random sample which included 450 village-level units in 150 counties (districts) in 28 provinces (autonomous regions and municipalities directly under the central government) in China (13). The CHARLS initially was approved by the ethical review committee of Peking University, and all informed consents were acquired from participants.

### Measurement of demographic characteristics

Participants were asked about their gender, date of birth, residency (rural or urban), specific address of residence (province/city/county), ethnicity (Han/ethnic minority), marital status (spouse/widowed/divorced/unmarried), education level (uneducated/not completed primary school/private school graduation/primary school graduation/junior high school graduation/high school graduation/junior college graduation/bachelor's degree/master's /PhD.), personal income, smoking status (yes/no), alcohol consumption status (yes/no), and religious status (yes/no).

### Health status measures

Participants were asked about their perception of current health status (Very good/Good/Fair average/Poor bad/Very poor), amount of sleep each night, whether they were physically disabled (yes/no) or mentally deficient (yes/no), whether they had insomnia or semi-insomnia (yes/no), whether they were deaf or semi-deaf (yes/no). Moreover, participants provided information on their social activities (socializing with friends, playing cards, volunteering, attending training), whether they exercised at least 10 min a week (yes/no), depression status, whether they had arthritis or rheumatism (yes/no), whether they had stomach problems (yes/no), and medical history (hypertension, high or low blood cholesterol, diabetes or elevated blood sugar, malignancy, chronic lung disorders, liver disease, heart disease, stroke or brain hemorrhage, kidney disease, stomach disease or digestive disorder, emotional and mental problems, memory-related disorders, arthritis or rheumatism, asthma).

### Health service utilization measures

Participants were asked about visits to various healthcare facilities, including Western medicine hospitals, specialty hospitals, Chinese medical hospitals, and other healthcare facility. Among these institutions, acupuncture and the use of Chinese herbal medicine in Chinese medical hospitals have been effective for chronic pain. Chinese medical hospitals treatment places great importance on the unity of the body itself and the relationship between the body and nature, and the Chinese people trust Chinese medical hospitals more than western medical hospital (14). In addition, they were asked about hospitalization during the past year.

### Outcome measures

Participants were asked, “Do you often have difficulty with pain?” The options were “not at all,” “a little,” “some,” “quite a lot” “very much.” If participants answered any of the “a little,” “some,” “more,” “very much” options, they were then asked, “Which parts of the body are painful? (Please list all parts).” These include head, shoulders, arms, wrists, fingers, chest, stomach, back, lower back, hips, legs, knees, ankles, toes, neck, and other areas.

### Statistical methods

A chi-square test was used to separately test differences in categorical measures between middle-aged and older adults with physical pain and those who did not have physical pain problems. Unconditional logistic regression models were developed to identify statistically significant factors associated with middle-aged and older adults with physical pain problems. All of the above demographic characteristics, health status and health service utilization that were statistically significant in the univariate analysis were entered into the model; a stepwise forward approach was then used to generate the most parsimonious model using a likelihood ratio test. In this study, the *p*-value threshold for statistical significance was <0.05 (15). All analyses were conducted using the statistical software Stata 16.

## Results

Over half of the respondents were female (51.3%), and the majority of participants were between 45 and 69 years (81.7%). Of the 15,423 aged 45 years and older, 9,257 (60.02%) had pain problems; among them, lower back (5,758, 62.2%), knees (4,369, 47.2%) and shoulders (4,092, 44.2%) were the three areas with the highest rate of body pain. Table 1 compares the demographic characteristics of middle-aged and older adults with and without pain problems, all these variables were statistically significantly different in the one-way analysis of variance. Among them, Middle-aged and older adults with pain were more likely to be female, those with less than a high school education were more likely to have pain and those with an income of US$1,000 or less were more likely to have pain.

**Table 1:** Comparison of physical pain rates in 15,423 Chinese older adults.

**Demographic characteristics**		**Number of cases *n* = 15,423 (%)**	**Body pain *n* = 9,257 (%)**	***X^2^***	***P*-value**
Gender	Male	7,506 (48.7)	3,856 (41.7)	455.80	<0.001
	Female	7,917 (51.3)	5,401 (58.3)		
Age	45~	7,290 (47.3)	42,65 (46.1)	14.12	<0.001
	60~	5,301 (34.4)	3,234 (34.9)		
	70	2,832 (18.3)	1,758 (19)		
Region	Eastern China	5,167 (33.5)	2,704 (29.2)	227.61	<0.001
	Central China	5,187 (33.6)	3,165 (34.2)		
	Western China	5,069 (32.9)	3,388 (36.6)		
Place of residence	Urban	6,305 (40.9)	5,754 (62.2)	88.47	<0.001
	Rural	9,118 (59.1)	3,503 (37.8)		
Ethnicity	Han Chinese	14,255 (92.4)	8,497 (91.8)	13.42	<0.001
	Ethnic minority	1,168 (7.6)	760 (8.2)		
Marital status	With spouse	13,514 (87.6)	8,013 (86.6)	24.16	<0.001
	other	1,909 (12.4)	1,244 (13.4)		
Education level	Never attended school	2,814 (18.3)	1,910 (20.6)	275.45	<0.001
	Did not finish elementary school	3,162 (20.5)	2,087 (22.5)		
	Elementary school	3,611 (23.4)	2,175 (23.5)		
	Middle School	3,697 (23.9)	2,055 (22.3)		
	High school and above	2,139 (13.9)	1,030 (11.1)		
Personal income	<1,000	11,500 (74.6)	7,264 (78.5)	227.72	<0.001
	1,000~	265 (1.7)	172 (1.9)		
	3,000~	350 (2.3)	211 (2.3)		
	6,000~	3,308 (21.4)	1,610 (17.3)		
Smoking status	Yes	4,683 (30.4)	2,580 (27.9)	68.11	<0.001
Alcohol consumption	Yes	5,519 (35.8)	2,991 (32.3)	121.63	<0.001
Religious status	Yes	1,495 (9.7)	963 (10.4)	13.32	<0.001

Moreover, Table 2 illustrates the contrasting health status between middle-aged and older adults with pain problems and those without pain problems. Of these, all but two variables, Dental problems and Social activity, were statistically significantly different between the two groups. Among them, people with physical disabilities, vision problems, hearing problems and arthritic conditions are more likely to suffer from pain.

**Table 2:** The relationship between physical pain and health status in middle-aged and older adults.

**Health status**		**Number of cases *n* = 15,423 (%)**	**Body pain *n* = 9,257 (%)**	***X^2^***	***P*-value**
Self-assessed health status	Very good	1,979 (12.8)	556 (6)	1,900.00	<0.001
	Good	2,016 (13.1)	851 (9.2)		
	Fair	7,675 (49.8)	4,741 (51.2)		
	Not good	2,889 (18.7)	2,369 (25.6)		
	Very bad	864 (5.6)	740 (8)		
Night sleep time	<7	8,704 (56.5)	5,772 (62.4)	332.22	<0.001
	7~	5,527 (35.8)	2,843 (30.7)		
	9~	1,192 (7.7)	642 (6.9)		
Physical disability	Yes	1,429 (9.3)	997 (10.8)	62.40	<0.001
Intellectual disability	Yes	1,137 (7.4)	847 (9.2)	107.21	<0.001
Vision problems	Yes	1,985 (12.9)	1,467 (15.8)	183.80	<0.001
Hearing problems	Yes	2,572 (16.7)	1,985 (12.9)	157.13	<0.001
Dental problems	Yes	120 (0.8)	80 (0.9)	2.20	0.14
Social activity	Yes	2,814 (18.3)	8,561 (92.5)	0.80	0.37
Depressive conditions	Yes	6,920 (44.9)	5,035 (54.4)	848.92	<0.001
Suffering from arthritis or rheumatism	Yes	5,914 (38.3)	4,592 (49.6)	1,200.00	<0.001
Suffering from stomach disorders	Yes	4,672 (30.3)	3,544 (38.3)	700.40	<0.001
Suffering from at least one chronic disease^*^	Yes	12,153 (21.2)	7,960 (86)	716.70	<0.001

* Suffering from at least one of the 14 chronic diseases: hypertension, dyslipidemia, high blood sugar, malignancy, lung disease, liver disease, heart disease, Stroke, Kidney disease, emotional and mental problems, memory-related disorders, and asthma.

Table 3 indicates that middle-aged and older adults with pain were more likely to visit the Western hospital, the Chinese medicine hospital, the specialty hospital, and other medical facility; and to be hospitalized within a year compared to middle-aged and older adults without pain.

**Table 3:** The relationship between physical pain and health service utilization.

**Health service utilization**		**Number of cases *n* = 15,423 (%)**	**Body pain *n* = 9,257 (%)**	***X^2^***	***P*-value**
Hospitalization within 1 year	Yes		1,755 (19)	178.26	<0.001
Visited a western hospital within 1 month	Yes	1,031 (6.7)	746 (8.1)	70.14	<0.001
Chinese medicine hospital visit	Yes	192 (1.2)	144 (1.6)	18.23	<0.001
Specialized hospital visit	Yes	115 (0.7)	83 (0.9)	7.18	0.01
Visits to other medical institutions^*^	Yes	1,342 (8.7)	1,026 (11.1)	165.41	<0.001
Social support	Yes	8,668 (56.2)	5,109 (55.2)	9.62	0.00

* Community health service centers, township health centers, older adults institutions, and private clinics.

Table 4 demonstrates statistically significant predictors of physical pain for middle-aged and older participants identified by the logistic regression model. The Hosmer and Lemeshow goodness-of-fit statistical value for this regression model was not significant (*R*^2^ = 18.03%, *p* = 0.368), referring to the percentage change of 18.03% for middle-aged and older adults who experienced the physical pain described by the model. Older female participants were more likely to be in pain compared to male participants (OR = 2.10). Those who reported that they were from the central region (OR = 1.15) and those from the western region (OR = 1.28) were more likely to have pain compared to those from the eastern region. Middle-aged and older adults living in rural areas (OR = 1.14) had a higher probability of having pain. The participants with smoking (OR = 1.26) and alcohol consumption (OR = 1.16) conditions were more likely to have pain. Compared to middle-aged and older adults who reported their general health as fair (OR = 2.30, 95% CI 2.67–3.37, *p* < 0.001), those who perceive their health as poor (OR = 6.05,), or very poor (OR = 6.84,) had a higher probability of having pain. Compared to middle-aged and older adults who reported that their nighttime sleep was 7 h and more but <9 h (OR = 0.74) or ≥9 h (OR = 0.69), the probability of pain was higher in middle-aged and older adults with <7 h of sleep. Those with hearing problems (OR = 1.23), depression (OR = 1.56), diagnosed arthritis or rheumatism (OR = 2.21) and stomach disorders (OR = 1.69) were more likely to have body pain in middle-aged and older adults. In addition, people with visits to Western medicine hospitals (OR =1 .28) and other medical facilities (OR = 1.42) were more likely to suffer from pain in middle-aged and older adults.

**Table 4:** Logistic regression to determine statistically significant predictors.

**Pain predictors**		**Odds ratio**	**95% CI**	***waldx^2^***	***p*-value**
Gender	Male	1.00	–		<0.001
	Female	2.10	1.90–2.33	205.92	
Region	Eastern China	1.00	–		<0.005
	Central China	1.15	1.05–1.25	9.06	
	Western China	1.28	1.16–1.41	26.21	
Place of residence	Urban	1.00	–		0.001
	Rural	1.14	1.06–1.23	10.89	
Smoking status	NO	1.00	–		<0.001
	Yes	1.26	1.14–1.38	21.53	
Alcohol consumption	NO	1.00	–		0.001
	Yes	1.16	1.06–1.26	10.69	
Self-assessed health status	Very good	1.00	–		<0.001
	Good	1.69	1.47–1.95	53.88	
	Fair	2.30	2.67–3.37	338.93	
	Not good	6.05	5.20–7.05	539.63	
	Very bad	6.84	5.41–8.65	259.21	
Night sleep time	<7	1.00	–		<0.001
	7~	0.74	0.68–0.80	57.76	
	9~	0.69	0.60–0.79	27.88	
Hearing problems	No	1.00	–		<0.001
	Yes	1.23	1.11–3.37	14.21	
Depressive conditions	No	1.00	–		<0.001
	Yes	1.56	1.03–1.29	125.89	
Suffering from arthritis or rheumatism	No	1.00	–		<0.001
	Yes	2.21	2.02–2.41	321.84	
Suffering from stomach disorders	No	1.00	–		<0.001
	Yes	1.69	1.55–1.85	135.50	
Visited a Western hospital within 1 month	No	1.00	–		0.002
	Yes	1.28	1.10–1.50	9.98	
Visits to other medical institutions^*^	No	1.00	–		<0.001
	Yes	1.42	1.22–1.64	21.62	

* Community health service centers, township health centers, older adults institutions, private clinics.

## Discussion

This study based on a large population-based survey explored the demographic characteristics, health status, and health service utilization of people aged 45 years and older in China. In this survey, 60.02% of the participants experienced pain and the data did not reflect an increase in the incidence of physical pain with age. However, other studies have found an increase in the prevalence of body pain with age (16).

Specifically, women are more likely to experience physical pain than men, which is consistent with previous studies (17, 18), and women may be experiencing more pain (19, 20). Thus, the findings provided evidence for targeted strategies by the health sector to address pain in middle-aged and older women.

This study found that physical pain was regionally related, with middle-aged and older adults in central and western China are more likely to suffer from physical pain than those in eastern China, suggesting that the prevalence of pain among middle-aged and older adults differs by region; This finding is similar to that of Jiang Yingying et al., who found that the prevalence of chronic pain varied between provinces in China (21). This finding supports developing region-specific policies to manage pain in middle-aged and older adults in China.

On the health behavior aspect, this survey study suggests that middle-aged and older adults who smoke and drink alcohol are more likely to experience physical pain, this finding is identical to the previous studies (22, 23). Furthermore, this finding from current study is consistent with the results of a cross-sectional study of 10,000 people in the general working population, indicating that smoking is associated with a higher risk of musculoskeletal pain (24). Therefore, smoking cessation and alcohol restriction can relieve pain in middle-aged and older adults.

This study demonstrated that participants' self-rated health status was strongly associated with physical pain. A previous study found that the worse the subjective health status of the respondents, the more likely they were to suffer from chronic back pain (25). It has also been suggested that patients with chronic pain are much more likely to choose not good than good for their self-rated health status, i.e., patients with pain are four to five times less likely to rate their health status as good than those without pain (26). In the present study, we were not able to clarify the causal relationship between self-rated health status and chronic pain, and we will need to explore this in more depth in the future.

Moreover, this study proposed that sleeping <7 h at night was a predictor of reporting physical pain in middle-aged and older adults. This is consistent with a recent Danish longitudinal study showing that the more severe the sleep disorder, the higher the pain level (27). Therefore, pain management and education for middle-aged and older adults should be accompanied by more attention to those with sleep disorders.

The results of this study suggest that the presence of hearing problems is a predictor of physical pain in middle-aged and older adults. Previous studies (28) have concluded that people with pain are more likely to fill in hearing impairment,; the two may be in an interactive relationship that needs to be further studied. Additionally, this study found that middle-aged and older adults suffering from depression were more likely to experience pain, which further supports the idea that the management and prevention of depression have great potential to improve physical pain in middle-aged and older adults (29).

Chronic pain is known to be one of the symptoms of gastric disorders, arthritis, and other chronic diseases (11, 30). This study showed that having stomach disorders and arthritis were predictors of reporting physical pain in middle-aged and older adults, which is consistent with the study by Lu Yang et al. (31). Considering that the burden of chronic diseases on health care systems will increase with the increasing aging population, it is necessary to provide relevant pain relief measures for patients with chronic diseases. At the same time, our health system should be continuously strengthened to provide better medical services. Pain management should be considered part of chronic diseases intervention strategies to improve patients' quality of life.

There is no doubt that a link exists between visits to healthcare institutions and physical pain, which is consistent with previous research (32) that chronic pain increases the burden on public health services and has a significant socioeconomic impact. This study found that older adults with physical pain were 1.28 times more likely to visit a Western hospital than older adults without physical pain, and 1.42 times more likely to visit other healthcare providers. In addition, a previous study found that a large number of patients choose to use drug injections for treatment, which has many potential risks (33). We need to clearly understand why, when, and how middle-aged and older patients with pain choose to go to a healthcare facility so that health providers and policymakers can better understand the reality of patients' situations and ensure more effective, comprehensive, and safe health services.

Although multiple findings are consistent with previous studies, this study has limitations. First, the data were obtained from the 2018 China Health and Aging Tracking Survey (CHARLS) database, which is a cross-sectional study, and the study could only examine the pain status of older adults patients at one point. Although the investigators have adjusted to include multiple indicators, However, the model does not include certain variables that may influence physical pain, such as genetic factors, and further research on this is needed in the future. Secondly, the data in this case are from a self-reported survey and the subjectivity of the respondents may have an impact on the veracity of the data. Moreover, the study utilizes a large proportion of older participants, who completed questionnaires with potentially recalled biased information. However, these limitations could be offset by the listed facts: the data for this study were extracted from a nationally representative and comprehensive sample from a large database that has been established for more than a decade and is part of a secondary data analysis that analyzes the demographic characteristics, health status, and health service utilization of middle-aged and older patients with pain. At the same time, this study only explored the prevalence and influencing factors of chronic pain in middle-aged and older adults people, and further research is needed in the future to investigate the relationship between single-site pain and multi-site pain.

## Conclusion

The purpose of this study is to provide a comprehensive determinant of demographic characteristics, health status, and health service utilization of middle-aged and older Chinese patients with physical pain from the public health domain, with the hope of helping healthcare providers to better prevent and manage pain for their patients and to encourage middle-aged and older patients with physical pain to disclose more information about pain in order to assess more influential factors of pain. In addition, study results provide health service decision-makers with more evidence about pain in middle-aged and older adults to develop better strategies for the prevention and management of physical pain. Meanwhile, further studies are needed to assess the factors associated with physical pain in middle-aged and older adults.

## Data availability statement

Publicly available datasets were analyzed in this study. This data can be found here: https://charls.charlsdata.com/pages/Data/2018-charls-wave4/zh-cn.html.

## Ethics statement

The studies involving human participants were reviewed and approved by Ethical Review Committee of Peking University. The patients/participants provided their written informed consent to participate in this study.

## Author contributions

ST led the research on this topic and provided guidance on all aspects of the design, writing, and revision of the article. ZA drafted the manuscript. All authors contributed to this article and approved the submission of the version.
